# Predictors of clinical features in early‐onset severe systemic sclerosis: An analysis from a multicenter prospective observational Japanese cohort

**DOI:** 10.1111/1346-8138.17403

**Published:** 2024-09-05

**Authors:** Saori Uesugi‐Uchida, Manabu Fujimoto, Yoshihide Asano, Hirahito Endo, Daisuke Goto, Masatoshi Jinnin, Yasushi Kawaguchi, Sumiaki Tanaka, Takahiro Tokunaga, Katsunari Makino, Takashi Matsushita, Sei‐Ichiro Motegi, Ayumi Yoshizaki, Shinichi Sato, Minoru Hasegawa

**Affiliations:** ^1^ Department of Dermatology University of Fukui Fukui Japan; ^2^ Department of Dermatology, Graduate School of Medicine Osaka University Osaka Japan; ^3^ Department of Dermatology Tohoku University Graduate School of Medicine Sendai Japan; ^4^ Department of Rheumatology Southern Tohoku General Hospital Fukushima Japan; ^5^ Department of Internal Medicine, Faculty of Medicine University of Tsukuba Ibaraki Japan; ^6^ Department of Dermatology Wakayama Medical University Graduate School of Medicine Wakayama Japan; ^7^ Institute of Rheumatology, Tokyo Women's Medical University Tokyo Japan; ^8^ Kitasato University Hospital Sagamihara Japan; ^9^ Medical Research Support Center University of Fukui Hospital Fukui Japan; ^10^ Research Promotion Office Shinseikai Toyama Hospital Toyama Japan; ^11^ Department of Dermatology and Plastic Surgery, Faculty of Life Sciences Kumamoto University Kumamoto Japan; ^12^ Department of Dermatology, Faculty of Medicine, Institute of Medical, Pharmaceutical and Health Sciences Kanazawa University Kanazawa Japan; ^13^ Department of Dermatology Gunma University Graduate School of Medicine Maebash Gunma Japan; ^14^ Department of Dermatology University of Tokyo Graduate School of Medicine Tokyo Japan; ^15^ Department of Clinical Cannabinoid Research University of Tokyo Graduate School of Medicine Tokyo Japan

**Keywords:** digital ulcer, health assessment questionnaire, lung, predictive factor, skin, systemic sclerosis

## Abstract

As the clinical course of systemic sclerosis (SSc) varies widely, prognostic indicators have been sought to predict the outcomes of individual patients. Racial differences in SSc render it necessary to validate prognostic indicators in different patient cohorts. In this study, we aimed to assess clinical and laboratory parameters in Japanese patients with early‐stage SSc with diffuse cutaneous involvement and/or interstitial lung disease, and identify predictive factors for disease progression. We performed multivariate analyses of baseline clinical information to estimate symptoms 4 years later in Japanese patients with diffuse cutaneous SSc and/or SSc with interstitial lung disease. Patients were enrolled in the study within 5 years of disease onset at 10 Japanese SSc centers. Over 12 years, 115 patients followed up for 4 years were included in this study. The modified Rodnan skin score (mRSS) at 4 years correlated with the baseline mRSS and finger‐to‐palm distance, defined as the average length from the distal tip of the fourth finger to the distal palmar crease. The percentage predicted vital capacity (%VC) in year 4 positively and negatively correlated with initial %VC and the presence of anti‐topoisomerase I antibodies, respectively. The Health Assessment Questionnaire Disability Index (HAQ‐DI) at 4 years was positively and negatively associated with baseline HAQ‐DI and %VC, respectively. The occurrence of digital ulcers within 4 years was associated with the initial presence of digital ulcers, finger‐to‐palm distance, and the presence of digital pitting scars and anti‐topoisomerase I antibodies. This study identified several factors that may predict the progression of early‐stage SSc in Japanese patients. Finger‐to‐palm distance may be a useful tool for predicting the progression of skin thickening and the development of digital ulcers in the early stages of severe SSc, but larger, long‐term prospective studies are needed to confirm our findings.

**Abbreviations**
%DLCO: percent predicted diffusion capacity of the lungs for carbon monoxide
%VC: percent predictive vital capacity
CI: confidence interval
dcSSc: diffuse cutaneous systemic sclerosis
ESR: erythrocyte sedimentation rate
EUSTAR: European Scleroderma Trials and Research
HAQ‐DI: Health Assessment Questionnaire Disability Index
ILD: interstitial lung disease
IQR: interquartile range
lcSSc: limited cutaneous systemic sclerosis
mRSS: modified Rodnan skin score
OR: odds ratio
PAH: pulmonary arterial hypertension
SSc: systemic sclerosis

## 1 INTRODUCTION

Systemic sclerosis (SSc) is a rheumatic disease that causes fibrosis of the skin and various internal organs as well as vasculopathy.^1^ SSc can result in digital ulcers and joint contractures due to notable skin sclerosis; extensive internal organ involvement can lead to potentially serious complications. Although various organs can be affected, lung involvement occurs in over half of patients with SSc, and lung damage is a principal cause of mortality.^2^

The majority of patients with SSc experience severe organ involvement within 3 years of onset, and rapid progression of skin sclerosis is not usually seen after the initial 5‐year disease period.^2^ The median peak modified Rodnan skin score (mRSS) reportedly occurs 4 months after initial presentation in patients with diffuse cutaneous involvement (dcSSc).^3^ However, there are no established biomarkers that can predict disease progression in patients with early‐stage SSc. Identification of potential biomarkers requires the consideration of racial variations in clinical and laboratory features, including autoantibody profiles, the severity of skin sclerosis, and the prevalence of organ lesion.^4^

Prognostic indicators of disease progression have been proposed in patients with SSc in previous studies.^5^, ^6^, ^7^, ^8^, ^9^ However, few attempts have been made to investigate the medium‐term course of SSc, especially in Japanese patients. It is therefore important to conduct observational studies on this population.

The present study aimed to assess clinical and laboratory parameters in the Japanese cohort with early‐stage SSc with dcSSc and/or interstitial lung disease (ILD) to identify factors that predict disease progression. We also identified factors that predicted the modified Rodnan skin score (mRSS), percentage predictive vital capacity (%VC), and Health Assessment Questionnaire Disability Index (HAQ‐DI) at 4 years, and digital ulcers occurring within 4 years.

## 2 METHODS

### 2.1 Patients

We used a multicenter prospective observational cohort of Japanese SSc patients, part of which had already been used in other studies, for the analysis.^10^, ^11^, ^12^ From January 2002 to April 2013, data on Japanese SSc cases (disease duration <5 years) who diagnosed as dcSSc and/or ILD were prospectively collected at the following 10 centers: Gunma University Hospital, Kanazawa University Hospital, Keio University Hospital, Kitasato University Hospital, Kumamoto University Hospital, Nagasaki University Hospital, Toho University Omori Medical Center, Tokyo University Hospital, Tokyo Women's Medical University Hospital, and Tsukuba University Hospital. All patients met the diagnostic criteria for SSc proposed by the American College of Rheumatology in 1980.^13^ According to the classification criteria proposed by LeRoy et al.,^14^ cases were classified into two clinical forms, limited cutaneous SSc (lcSSc) and dcSSc, based on the extent of skin thickening. The study was approved by the ethics committee of each participating center, and written informed consent was available in all patients.

### 2.2 Clinical assessments

Participants underwent physical examinations and laboratory tests at their respective registration centers on enrolment and were subsequently followed up for 4 consecutive years, with clinical and laboratory parameters measured at least once a year.

**FIGURE 1:**
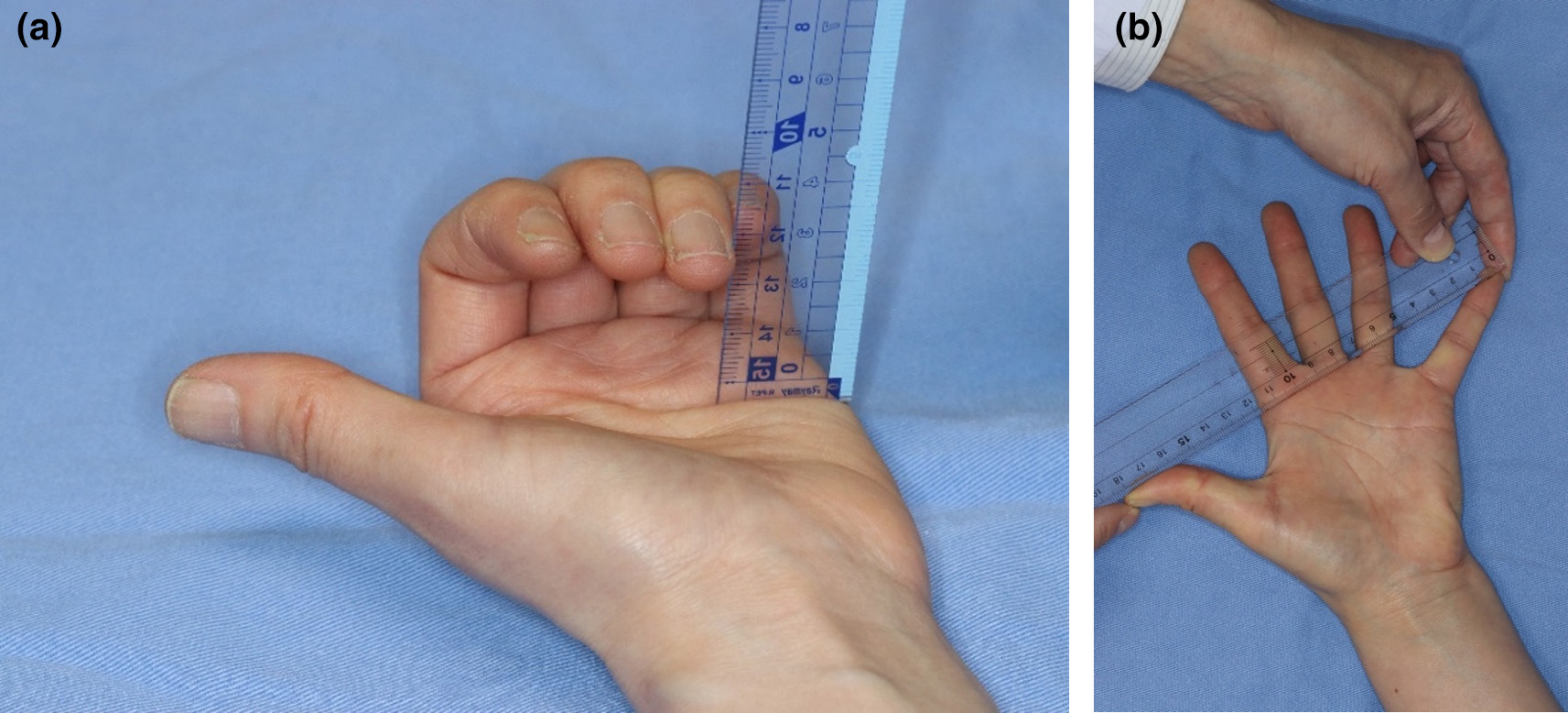
(a) Finger‐to‐palm distance, defined as the distance from the distal tip of the fourth finger to the distal palmar crease. (b) Hand‐extension, defined as the distance from the furthest point of the thumb to the furthest point of the fifth finger during maximum finger extension.

The degree and severity of skin involvement were determined by calculating the mRSS, as previously described.^15^ Each organ involvement was evaluated with modification of the previous report^2^: ILD was diagnosed by the presence of bilateral interstitial fibrosis or ground‐glass shadows on high‐resolution computed tomography, clinically suspected pulmonary arterial hypertension (PAH) was recorded when there was clinical manifestations of pulmonary hypertension and elevated right ventricular systolic pressure (>45 mmHg) detected by echocardiography in the absence of severe lung fibrosis, and joint involvement was defined as the presence of inflammatory polyarthralgia and/or arthritis. Reflux/dysphagia symptoms and the presence of lower gastrointestinal symptoms such as bowel obstruction and malabsorption syndrome were checked. However, since none of the patients experienced lower gastrointestinal symptoms during the 4‐year period, only reflux/dysphagia symptoms were used in this analysis. At initial enrollment, no patients had scleroderma renal crisis. Four patients developed renal crisis within 4 years, but the number was so small that it was not included in the analysis. An HAQ‐DI modified for Japanese patients was completed by all participants,^16^ and the incidences of digital ulcers (occurred within the past year) and digital pitting scars were recorded. Regarding digital ulcers, ulcers on the fingertips caused by blood flow disturbance were included, and ulcers on the dorsal surface of the finger joints by physical irritation or other causes were excluded. The oral aperture (the length from the base of the upper lip to the top of the lower lip when the mouth is fully open) was measured three times in each participant,^17^ and the finger‐to‐palm distance, also called active fist closure or the finger flexion distance (the length from the distal tip of the fourth finger to the distal palmar crease) (Figure 1a),^17^, ^18^ and hand‐extension, also called the hand spread (defined as the length from the furthest point of the thumb to the furthest point of the fifth finger during maximum finger extension) (Figure 1b),^17^, ^18^ were also determined. Pulmonary function was assessed by measuring the percentage predicted vital capacity (%VC) and the percentage predicted diffusion capacity of the lungs for carbon monoxide (%DLCO). The formula for %DLCO and the adjustment of %DLCO for hemoglobin levels differed among centers. Organ dysfunction data were not statistically adjusted for cases whose organ function was not evaluated, and the incidence rates were calculated only for those assessed for organ dysfunction.

### 2.3 Statistical analysis

All statistical analyses were performed using IBM SPSS Statistics for Windows, version 28.0 (IBM Corporation, Armonk, NY, USA). Missing values at the first visit were replaced with multiple imputations. Continuous variables are expressed as median and interquartile range (IQR), and categorical variables are expressed as numbers and percentages. Factors associated with the mRSS, %VC, and HAQ‐DI were identified using multiple linear regression analysis, whereas factors associated with digital ulcers were determined using multiple logistic regression analysis. Before performing regression analysis, the normality of the variables was confirmed using the Shapiro–Wilk test; no variables deviated significantly from the normal distribution. In addition, we determined the Pearson's correlation coefficient and found no variables with correlation coefficients >0.9. The results of the multiple regression analyses are expressed as odds ratios (ORs) and 95% confidence intervals (CIs). Statistical significance was set at *P* < 0.05.

## 3 RESULTS

### 3.1 Baseline patient characteristics

During the 12‐year study period, a total of 207 Japanese patients were included in this study. Of these, 115 were followed up for at least 4 years from the initial visit; these 115 patients were all included in the analysis and were assessed once a year for 4 years.

The baseline patient characteristics are summarized in Table 1. Of the 115 patients, 87 (75.7%) were female and 28 (24.3%) were male; the median age was 52.0 years (IQR 39.50–59.00 years). The median disease duration at the first visit was 22 months (IQR 9.50–36.00 months). A total of 90 patients had dcSSc, 56 with ILD, 27 without, and seven without ILD data availability; 25 patients had lcSSc. Anti‐topoisomerase I antibody was positive for 73 patients (63.5%), and anti‐centromere antibody was for 14 patients (12.2%). We could not evaluate the presence of anti‐RNA polymerase III or anti‐U1 RNP antibodies owing to limitations in the available data.

**TABLE 1:** Patient characteristics at baseline (*n* = 115).

Characteristic	*n* (%) or median (IQR)
Sex, *n* (%)	
Female	87/115 (75.7)
Male	28/115 (24.3)
Age at onset (years), median (IQR)	52 (39.5–59.0)
Disease duration (months), median (IQR)	22 (9.5–36.0)
Disease phenotype, *n* (%)	
dcSSc	90/115 (78.3)
lcSSc with ILD	25/115 (21.7)
Antibody detected, *n* (%)	
Anti‐centromere	14/115 (12.2)
Anti‐topoisomerase I	73/115 (63.5)
mRSS, median (IQR)	19 (11.0–24.0)
ESR (mm/h), median (IQR)	18 (9.5–30.0)
Maximal oral aperture (mm), median (IQR)	45 (40.0–50.0)
Finger‐to‐palm distance (mm), median (IQR)	2 (0.0–14.8)
Hand‐extension (mm), median (IQR)	175 (159.5–190.0)
Digital gangrene, *n* (%)	0/115 (0)
Digital ulcer, *n* (%)	20/115 (17.4)
Pitting scar, *n* (%)	38/112 (33.9)
Nailfold bleeding, *n* (%)	81/113 (71.7)
Joint involvement, *n* (%)	25/115 (21.7)
PAH, *n* (%)	14/111 (12.6)
ILD, *n* (%)	81/104 (77.8)
%VC, median (IQR)	96.4 (85.0–107.7)
%DLCO, median (IQR)	70.6 (57.1–82.7)
Reflux/dysphagia symptoms, *n* (%)	49/115 (42.6)
HAQ‐DI, median (IQR)	0.125 (0.0–0.5)

Abbreviations: %DLCO, percentage predicted diffusing capacity of the lungs for carbon monoxide; %VC, percentage predicted vital capacity; dcSSc, diffuse cutaneous systemic sclerosis; ESR, erythrocyte sedimentation rate; HAQ‐DI, Health Assessment Questionnaire Disability Index; ILD, interstitial lung disease; IQR, interquartile range; lcSSc, limited cutaneous systemic sclerosis; mRSS, modified Rodnan skin score; PAH, pulmonary arterial hypertension.

### 3.2 Prognostic factors associated with skin sclerosis progression

We assessed the clinical and laboratory factors recorded at the first visit to identify factors that could predict the severity of skin thickening 4 years later. The initial median mRSS was 19, which improved to 9, 4 years later (Table 2). Based on published literature and expert opinion, the following 16 variables, measured at registration, were selected for multiple linear regression analysis to assess the physical ability to predict the mRSS 4 years later: sex, age at onset, disease duration, mRSS, %VC, maximal oral aperture, finger‐to‐palm distance, erythrocyte sedimentation rate (ESR), the presence of anti‐topoisomerase I antibodies, anti‐centromere antibodies, ILD, joint lesions, digital ulcers, digital pitting scars, and nailfold bleeding, and immunosuppressive treatment with prednisolone, intravenous cyclophosphamide, azathioprine, methotrexate, or cyclosporin A.

**TABLE 2:** Disease progression over the 4‐year study period.

	Baseline	Year 1	Year 2	Year 3	Year 4
mRSS, median (IQR), *n*	19.0 (11.0–24.0), *n* = 115	12.0 (7.0–17.0), *n* = 106	10.0 (5.0–16.0), *n* = 98	8 (4.0–16.0), *n* = 110	9.0 (3.0–16.0), *n* = 115
%VC, median (IQR), *n*	96.40 (85.00–107.70), *n* = 81	96.65 (80.80–109.475), *n* = 50	99.60 (78.10–109.10), *n* = 59	91.40 (78.425–108.525), *n* = 60	91.00 (77.60–104.30), *n* = 63
HAQ‐DI, median (IQR), *n*	0.125 (0.0–0.5), *n* = 115	0.125 (0.0–0.5), *n* = 113	0.125 (0.0–0.5), *n* = 98	0.125 (0.0–0.375), *n* = 108	0.125 (0.0–0.4687), *n* = 112
Digital ulcer, *n* (%)	20/115 (17.4)	24/101 (23.8)	18/94 (19.1)	20/107 (18.7)	27/111 (24.3)

Abbreviations: HAQ‐DI, health assessment questionnaire disability index; IQR, interquartile range; mRSS, modified Rodnan skin score; %VC, percentage predicted vital capacity.

Table 3 shows the results of this multiple regression analysis. Stepwise multiple regression predicted the mRSS at year 4 using the following formula: 4.34 + 0.231 × baseline mRSS + 0.204 × initial finger‐to‐palm distance (mm), which correlated well with baseline mRSS and initial finger‐to‐palm distance (*P* = 0.003 and *P* = 0.002, respectively). Thus, the baseline mRSS and initial finger‐to‐palm distance were significantly associated with the mRSS 4 years after the initial visit.

**TABLE 3:** Factors predicting mRSS after 4 years detected in multiple regression analysis.

Variable	Regression coefficient	95% CI	*P* value
Initial mRSS	0.231	0.080–0.383	0.003*
Initial finger‐to‐palm distance	0.204	0.073–0.335	0.002*

Abbreviations: CI, confidence interval; mRSS, modified Rodnan skin score.

* *P* < 0.05.

### 3.3 Prognostic factors associated with %VC

Similarly, we assessed the factors that predicted %VC 4 years after the initial visit using multiple regression analysis. The median %VC was 96.4 at initial enrolment and decreased to 91.0% 4 years later (Table 2). Based on published literature and expert opinion, the following 17 variables, measured at registration, were selected for multiple linear regression analysis: sex, age at onset, disease duration, mRSS, %VC, %DLCO, ESR, the presence of anti‐topoisomerase I antibodies, anti‐centromere antibodies, ILD, PAH, joint lesions, digital ulcers, digital pitting scars, nailfold bleeding, reflux/dysphagia symptoms, and the use of immunosuppressive treatment.

Table 4 shows the results of this multiple regression analysis. %VC at the 4th year was predicted using the following formula: 19.256 + 0.797 × baseline %VC – 0.6785 × presence of anti‐topoisomerase I antibodies (*P* < 0.001). Although the presence of anti‐topoisomerase I antibodies had a *P* value of 0.068, it was included in the model because it improved the fit of the final multivariate model, increasing the adjusted *R* ^2^ value. %VC at the 4th year was positively associated with baseline %VC and negatively associated with the presence of anti‐topoisomerase I antibodies.

**TABLE 4:** Factors predicting %VC after 4 years detected in multiple regression analysis.

Variable	Regression coefficient	95% CI	P value
Initial %VC	0.797	0.603–0.992	<0.001*
Anti‐topoisomerase I antibody	−6.785	−14.084–0.514	0.068

Abbreviations: CI, confidence interval; %VC, percentage predicted vital capacity.

* *P* < 0.05.

### 3.4 Prognostic factors associated with HAQ‐DI

We also assessed the factors that predicted the HAQ‐DI 4 years after the initial visit using multiple regression analysis. The median HAQ‐DI score was 0.125 at the initial enrolment; this value remained constant over the 4‐year study period (Table 2). Based on published literature and expert opinion, the following 18 variables, measured at registration, were selected for multiple linear regression analysis: sex, age at onset, disease duration, HAQ‐DI, mRSS, %VC, maximal oral aperture, finger‐to‐palm distance, ESR, the presence of anti‐topoisomerase I antibodies, anti‐centromere antibodies, ILD, PAH, joint lesions, digital ulcers, digital pitting scars, nailfold bleeding, and the use of immunosuppressive treatment.

Table 5 shows the results of this multiple regression analysis. HAQ‐DI at the 4th year was predicted using the following formula: 0.774 + 0.562 × baseline HAQ‐DI – 0.006 × baseline %VC (*P* < 0.001 and *P* = 0.016, respectively). Although baseline %VC had a *P* value of 0.016, it was included in the model because it increased the adjusted *R* ^2^ value of the whole model. HAQ‐DI at the 4th year significantly was positively associated with baseline HAQ‐DI and negatively associated with baseline %VC.

**TABLE 5:** Factors predicting HAQ‐DI after 4 years detected in multiple regression analysis.

Variable	Regression coefficient	95% CI	*P* value
Initial HAQ‐DI	0.494	0.299 to 0.689	<0.001*
Initial %VC	−0.007	−0.013 to −0.001	0.016*

Abbreviations: CI, confidence interval; HAQ‐DI, health assessment questionnaire disability index; %VC, percentage predicted vital capacity.

* *P* < 0.05.

### 3.5 Prognostic factors associated with the occurrence of digital ulcers

In addition, we investigated factors that predicted the occurrence of digital ulcers within 4 years of the initial visit using multiple logistic regression analysis. The incidence of digital ulcers was 17.4% at baseline and 24.3% after 4 years (Table 2). Based on published literature and expert opinion, the following 15 variables, measured at registration, were selected for regression analysis: sex, age at onset, disease duration, mRSS, %VC, HAQ‐DI, finger‐to‐palm distance, ESR, the presence of anti‐topoisomerase I antibodies, anti‐centromere antibodies, ILD, PAH, digital ulcers, digital pitting scars, and nailfold bleeding.

Of the 115 participants, 76 were taking prostaglandin preparations at registration, 102 were by the 4th year, and the remaining 13 were not. In addition, 14 participants were using calcium channel blockers at registration. As individuals who already had digital ulcers were more likely to use these peripheral vasodilators than individuals who had no ulcers, treatment with vasodilators was not included as a variable in this logistic regression analysis.

At the time of this study, the treatment of digital ulcers in patients with SSc with bosentan hydrate was not covered by insurance, therefore only one participant was using bosentan hydrate at registration. Three participants used bosentan hydrate at 1 year; this number increased to 12 at the 4th year.

We first performed logistic regression analysis using the likelihood ratio method to select the relevant variables; the forced entry method was used for the analysis. The results of the chi‐squared test were considered significant at *P* < 0.01. Table 6 shows the ORs and 95% CIs from the logistic regressions analysis. The presence of digital ulcers at the initial visit or within the past year (OR = 3.927, 95% CI 1.145–13.466, *P* = 0.030), baseline finger‐to‐palm distance (OR = 1.043, 95% CI 1.002–1.085, *P* = 0.040), the presence of digital pitting scars at initial visit (OR = 2.113, 95% CI 0.783–5.706, *P* = 0.140), and the presence of anti‐topoisomerase I antibodies (OR = 1.871, 95% CI 0.711–4.921, *P* = 0.205) were associated with an increased risk of digital ulcers occurring within 4 years of the initial visit.

**TABLE 6:** Factors predicting the development of digital ulcers after 4 years detected in multiple logistic regression analysis.

Variable	Odds ratio	95% CI	*P* value
Digital ulcers at registration	3.927	1.145–13.466	0.03*
Initial finger‐to‐palm distance	1.043	1.002–1.085	0.04*
Pitting scars at registration	2.113	0.783–5.706	0.14
Anti‐topoisomerase I antibody	1.871	0.711–4.921	0.205

Abbreviations: CI, confidence interval.

* *P* < 0.05.

## 4 DISCUSSION

This multicenter observational study of 115 patients with SSc aimed to examine prognostic factors for skin sclerosis, lung function, a decline in physical function, and the occurrence of digital ulcers during the first 4 years of follow‐up. We evaluated predictors of the mRSS, %VC, and HAQ‐DI 4 years after the initial visit using multiple regression analysis. Furthermore, we evaluated the prognostic factors for digital ulcers occurring after 4 years of the initial visit using multiple logistic regression analysis.

We found a positive correlation between the mRSS at the 4th year and baseline mRSS and finger‐to‐palm distance. A multicenter prospective observational study investigating SSc within 2 years of onset in the USA showed that 71% of patients classified as being at risk of progression to dcSSc at baseline developed dcSSc. These patients were characterized by a higher mean HAQ‐DI and a higher mRSS at baseline than those who did not progress from lcSSc.^19^ In contrast to this study and our results, other studies have identified low baseline mRSS as a potential predictor of progressive skin fibrosis.^5^, ^6^, ^7^ A possible reason for this is that, while our study predicted the mRSS at 4 years, previous studies^5^, ^6^, ^7^ divided patients into a skin thickness‐progressed group and an improved group based on the mRSS at 1 or 2 years. When predicting short‐term changes in skin sclerosis, a low baseline mRSS can be a predictor of worsening skin fibrosis; in contrast, the present study analyzed mRSS in the 4th year in a cohort in which most cases had already progressed to dcSSc at enrolment.

Finger‐to‐palm distance is a measurement of the distance between the fingertips and palm.^17^, ^18^ The normal value is 0 mm, which gradually increases with progressive joint flexion contracture and limited flexion of the metacarpophalangeal joint. In patients with dcSSc, the finger‐to‐palm distance increases during periods of rapid skin stiffening and remains steady or improves slowly subsequently,^17^ therefore an increase in this distance early in the disease onset is associated with subsequent skin thickening. Few reports have examined the usefulness of this measurement and it may therefore warrant further investigation.^20^

Another finding of our study was that %VC in the 4th year was positively associated with baseline %VC and negatively associated with the presence of anti‐topoisomerase I antibodies. These findings are consistent with those of previous studies that showed that lower forced vital capacity predicted ILD progression.^21^, ^22^ Moreover, Nihtyanova et al. hypothesized that the presence of anti‐topoisomerase I antibodies could predict a decline in %VC; this hypothesis is supported by our results.^22^ Anti‐topoisomerase I antibodies are associated with a higher prevalence and greater severity of ILD.^23^ These parameters can be easily measured in daily clinical practice and may be useful for predicting ILD. However, our results on predicting %VC contrast with other studies that have identified male sex, the presence of reflux/dysphagia symptoms, and a high baseline mRSS as risk factors for ILD progression in patients with SSc.^3^, ^8^ These variables were included in our study but did not improve the fit of our multivariable %VC prediction model. These differences may be explained by racial differences and the inclusion of only severe cases of SSc involving the skin and/or lungs. However, our study is limited by the small sample size, especially regarding %VC, therefore further research is needed to confirm our results.

The HAQ‐DI at 4 years showed positive and negative associations with the initial HAQ‐DI and %VC, respectively. ILD is the most common cause of death in patients with SSc and its severity has a significant impact on the quality of life of patients.^24^ The phase III SENSCIS trial investigating the utility of nintedanib in SSc‐ILD also showed that improvement or worsening of SSc‐ILD significantly correlated with health‐related quality of life changes, including those measured by the HAQ‐DI.^25^ An analysis of the European Scleroderma Trials and Research (EUSTAR) database identified the baseline HAQ‐DI score, corticosteroid treatment, and major advanced organ involvement as predictors of death in patients with SSc. The HAQ‐DI and major advanced organ disease reportedly have comparable predictive ability.^26^ The European Scleroderma Observational Study found that a higher HAQ‐DI was significantly associated with a higher mRSS. Moreover, the HAQ‐DI was higher in patients with digital ulcers, lung fibrosis, heart lesions, and muscle lesions at baseline.^27^ Baseline mRSS and baseline HAQ‐DI have been shown to be predictors of HAQ‐DI progression at 1 year.^26^ This study differs from ours in that respiratory function tests were not included in the analysis of predictors of HAQ‐DI and the analysis took place at 1 year, rather than 4 years.

We also evaluated risk factors for the initial appearance of a digital ulcer within 4 years. The presence of digital ulcers at first enrolment and finger‐to‐palm distance were significant risk factors. Digital pitting scars and the presence of anti‐topoisomerase I antibodies were also suggested to increase the risk of digital ulcers, although not significantly. In a study of the EUSTAR database with a median follow‐up of 2 years, the presence of PAH and multiple digital ulcers at diagnosis increased the risk of subsequent digital ulcer occurrence.^28^ An Australian study with a mean follow‐up period of 5.2 years identified diffuse skin sclerosis, the presence of anti‐topoisomerase I antibodies, a younger age at onset, and reduced health‐related quality of life as risk factors for digital ulcers.^9^ As the majority of cases in our cohort were dcSSc and PAH was rare, associations with skin thickening or PAH may have been difficult to distinguish. Almost no studies have assessed the finger‐to‐palm distance as a predictive factor. Patients with long finger‐to‐palm distances are typically unable to fully extend their fingers, which may contribute to ulcer formation due to impaired blood flow to the fingertips.

We previously assessed predictors of the mRSS and %VC at 3 years in a similar but smaller Japanese cohort.^10^ In that study, baseline mRSS and maximal oral aperture were identified as significant predictors, with positive and negative correlation, respectively, for the mRSS at 3 years. The baseline ESR also tended to correlate with the mRSS at 3 years. These results may differ slightly from those of the present study because of the differences in the sample size and follow‐up periods, and because the finger‐to‐palm distance was not included in the previous analysis. The present study revealed that the finger‐to‐palm distance is more useful for predicting the mRSS than the maximal oral aperture or ESR. In our previous study, %VC in the 3rd year was significantly associated with baseline %VC.^10^ Moreover, patients with anti‐topoisomerase I antibodies exhibited reduced %VC after 3 years. These findings were confirmed in the present study, which was expanded in both sample size and follow‐up duration.

This study has some limitations. Although the nailfold video capillaroscopy findings, especially the reduction of capillaries, and its correlation with the subsequent progression of skin sclerosis, the appearance and/or progression of organ lesions, and the development of digital ulcers^29^ has been recently reported, the capillaroscopy findings could not be included in this study because of lack of enough data. The number of cases was small, and a larger study is needed to confirm the predictors of early severe SSc in Japan. It may also be necessary to examine all patients with early SSc since there may be cases that progress even if they are not severe in the early phase. Treatment strategies varied between centers and cases, and as the study period was some time ago, the impact of recent treatments cannot be determined. We are currently resuming a similar multicenter registry that should soon identify changes in symptom progression and predictors with treatment shifts in Japanese patients with SSc.

## 5 CONCLUSIONS

In summary, the mRSS in year 4 correlated with the initial mRSS and finger‐to‐palm distance. The %VC at year 4 was positively and negatively associated with baseline %VC and the presence of anti‐topoisomerase I antibodies, respectively. The HAQ‐DI in year 4 was positively and negatively associated with the initial HAQ‐DI and %VC, respectively. The incidence of digital ulcers within 4 years was associated with the initial finger‐to‐palm distance and the presence of digital ulcers, digital pitting scars, and anti‐topoisomerase I antibodies. The finger‐to‐palm distance may be a useful technique for predicting not only the mRSS, but also the occurrence of digital ulcers.

## FUNDING INFORMATION

This work was supported by funds for research on intractable diseases from the Ministry of Health, Labor, and Welfare of Japan.

## CONFLICT OF INTEREST STATEMENT

None declared.

## Data Availability

The datasets generated and/or analyzed during the current study are available from the corresponding author on reasonable request.
